# The Iron Chelator, Dp44mT, Effectively Inhibits Human Oral Squamous Cell Carcinoma Cell Growth in Vitro and in Vivo

**DOI:** 10.3390/ijms17091435

**Published:** 2016-08-31

**Authors:** Jehn-Chuan Lee, Kun-Chun Chiang, Tsui-Hsia Feng, Yu-Jen Chen, Sung-Ting Chuang, Ke-Hung Tsui, Li-Chuan Chung, Horng-Heng Juang

**Affiliations:** 1 Department of Otolaryngology, Mackay Memorial Hospital, Taipei 105, Taiwan; entdrlee4606@gmail.com; 2 School of Medicine, Mackay Medical College, New Taipei City 207, Taiwan; 3 Zebrafish Center, Department of General Surgery, Chang Gung Memorial Hospital and University, Keelung 204, Taiwan; robertviolet6292@yahoo.com.tw; 4 School of Nursing, College of Medicine, Chang Gung University, Kwei-Shan, Tao-Yuan 333, Taiwan; thf@mail.cgu.edu.tw; 5 Department of Radiation On Cology, Mackay Memorial Hospital, Taipei 105, Taiwan; chenmdphd@gmail.com; 6 Department of Anatomy, College of Medicine, Chang Gung University, 259 Wen-Hua 1st Road, Kwei-Shan, Tao-Yuan 333, Taiwan; tingsj1223@gmail.com; 7 Department of Urology, Chang Gung Memorial Hospital-Linkou, Kwei-Shan, Tao-Yuan 244, Taiwan; t2130@adm.cgmh.org.tw; 8 Department of General Education Center, Mackay Medicine, Nursing and Management College, New Taipei City 207, Taiwan

**Keywords:** NDRG1, NDRG3, Maspin, DFO, deferasirox

## Abstract

Oral squamous cell carcinoma (OSCC) is a common malignancy with a growing worldwide incidence and prevalence. The *N*-myc downstream regulated gene (NDRG) family of NDRG1, 2, 3, and mammary serine protease inhibitor (Maspin) gene are well-known modulators in the neoplasia process. Current research has considered iron chelators as new anti-cancer agents; however, the anticancer activities of iron chelators and their target genes in OSCC have not been well investigated. We showed that iron chelators (Dp44mT, desferrioxamine (DFO), and deferasirox) all significantly inhibit SAS cell growth. Flow cytometry further indicated that Dp44mT inhibition of SAS cells growth was partly due to induction of G1 cell cycle arrest. Iron chelators enhanced expressions of NDRG1 and NDRG3 while repressing cyclin D1 expression in OSCC cells. The in vivo antitumor effect on OSCC and safety of Dp44mT were further confirmed through a xenograft animal model. The Dp44mT treatment also increased Maspin protein levels in SAS and OECM-1 cells. NDRG3 knockdown enhanced the growth of OECM-1 cells in vitro and in vivo. Our results indicated that NDRG3 is a tumor suppressor gene in OSCC cells, and Dp44mT could be a promising therapeutic agent for OSCC treatment.

## 1. Introduction

Oral squamous cell carcinoma (OSCC) accounts for 90% of all oral cancers, which are the most commonly diagnosed malignancies, estimated at over 300,000 new cases annually around the world [1,2]. Apart from the increasing incidence rates in many countries, the higher recurrence rate and poor prognosis are the primary reasons to identify new molecular targets and therapeutic agents for the management of OSCC [3,4].

Iron is an important trace element involved in a variety of cellular processes, including energy generation, oxygen transport, and DNA synthesis [5]. The higher iron demand in cancer cells due to rapid DNA synthesis and growth than in normal cells has implied the antitumor potential of iron chelators [6]. Three effective iron chelators, namely, di-2-pyridylketone 4,4-dimethyl-3-thiosemicarbazone (Dp44mT), desferrioxamine (DFO), and deferasirox, applied as medical treatment for iron overload disease, have been shown to possess antitumor activity for certain cancers [5,6,7,8,9]. Prior studies regarding Dp44mT, DFO, and deferasirox have demonstrated that the inhibitory effects on cancer cell proliferation and metastasis of these three drugs were mediated by several pathways, such as increasing levels of apoptosis markers including p21, p53, and activation of mitogen-activated protein kinases (MAPKs), and decreasing levels of cyclins and cyclin-dependent kinase 2 (CDK2) [8,10,11].

*N*-myc downstream regulated genes (NDRG) 1, 2, and 3 have been shown to play vital roles in the control of cell proliferation, differentiation, development, and stress response in human tissues [12,13]. However, unlike *NDRG1* and *NDRG2*, which are widely known as tumor suppressor genes, the role of NDRG3 in cancer is still inconclusive [14]. It has been shown that NDRG1 expression is inversely correlated with the mortality rate of OSCC patients, and overexpressed NDRG1 inhibited cell proliferation and tumorigenesis in OSCC cells [15,16]. NDRG2 overexpression was also shown to inhibit OSCC cell growth [17]. *NDRG1* and *NDRG2* have been further shown to be iron chelator responsive genes in a number of cancer cell lines [8,10,18,19,20]. Regarding *NDRG3*, although some studies reported that *NDRG3* might be an oncogene in prostate and liver cancers [21,22,23], the expressions and functions of *NDRG3* gene in OSCC cells have yet to be investigated.

Maspin (mammary serine protease inhibitor), a member of the serine protease inhibitor/non-inhibitor superfamily, has been shown to have tumor growth suppression activity in vitro and in vivo for a variety of cancers [24]. It has been reported that higher Maspin expression was associated with better overall survival rate and lower rate of lymph node metastasis; in contrast, in the OSCC patients, lower expression of Maspin was accompanied with larger tumors [25,26,27]. These findings suggest that Maspin could be a potential target in OSCC treatment.

The objectives of this study were to investigate the potential of iron chelator application for OSCC treatment and the effects of iron chelators on gene expressions of *NDRG1*, *NDRG2*, *NDRG3*, and *Maspin*. In addition, the role of *NDRG3 in OSCC cells was also be studied.*

## 2. Results

### 2.1. Dp44mT, Desferrioxamine (DFO), and Defrasirox Inhibited SAS Cell Growth

**Figure 1:**
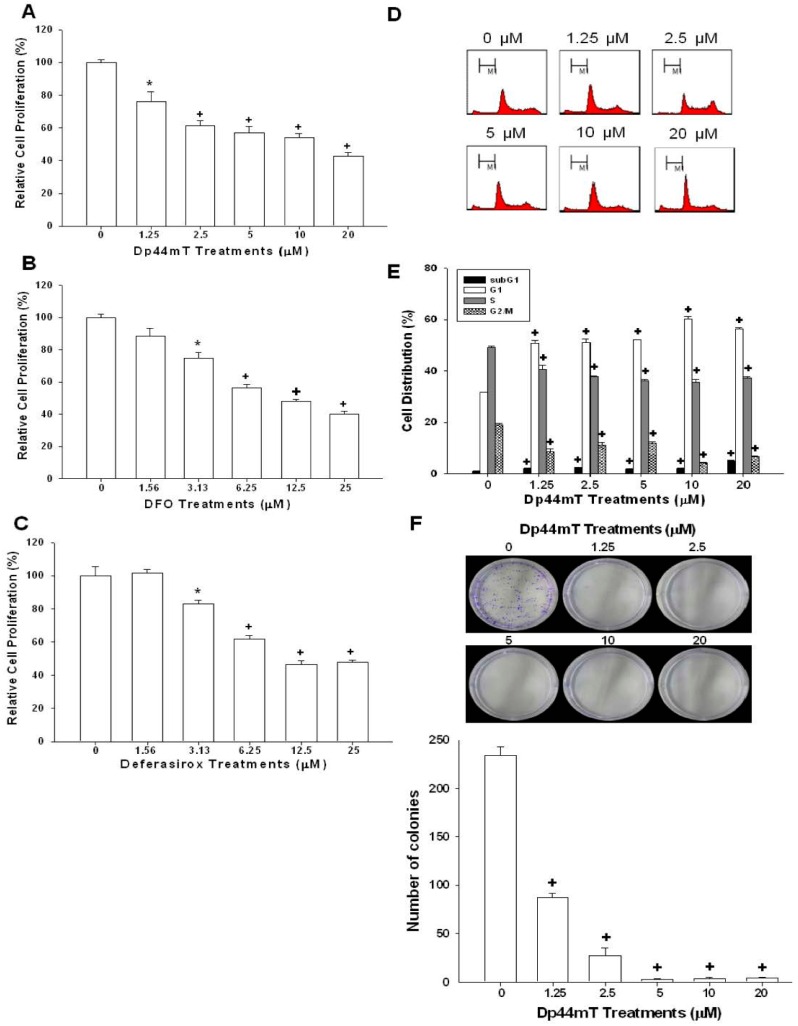
Anti-proliferative effects of Dp44mT, desferrioxamine (DFO), and deferasirox in SAS cells. The SAS cells were treated with various concentrations of Dp44mt (**A**), DFO (**B**), and deferasirox (**C**) as indicated for 24 h, and growth inhibitory effect was determined by the CyQUANT cell proliferation assay. The data shown in each bar chart represented the mean percentage ± SE of cells in each dose of the iron chelator treatment and were compared with the control solvent-treated group (*n* = 8); (**D**) SAS cells were treated with various concentrations of Dp44mT (0–20 µM). The cell cycle distribution of SAS cells was analyzed after 24 h of incubation by flow cytometry; (**E**) The data shown in each bar chart represented the mean percentage ± SE of cells in each phase of the cell cycle and were compared with the control solvent-treated group (*n* = 3); (**F**) SAS cells were treated with various concentrations of Dp44mT as indicated for 24 h and growth inhibitory effect was determined by the colony formation assay after incubation in RPMI medium with 10% FCS for another 7 days. The data shown in each bar chart represent the total numbers of clone ± SE (*n* = 5). * *p* < 0.05, + *p* < 0.01.

The CyQUANT cell proliferation assay reveals that Dp44mT, DFO, and deferasirox significantly inhibited the growth of SAS cells in a dose-dependent manner (Figure 1A–C). The EC50 for Dp44mT, DFO, and deferasirox is 27.8, 28.9, and 21 µM, respectively. The flow cytometric analysis reveals that Dp44mT induced cell cycle arrest at G1 phase dose-dependently after 24 h of treatments in SAS cells. There was a 19.5%–28.5% increase in G1 phase cells under 1.25–10 µM Dp44mT treatment, together with a decrease of cells at S and G2/M phases in SAS cells (Figure 1D,E). The colony formation assay also shows that Dp44mT significantly inhibited the reproductive viability of SAS cells (Figure 1F). We further tested Dp44mT′s effect on normal epithelial cells and HaCaT cells. As shown in the supplemental data, DP44mT still could inhibited HaCaT cell growth, but the inhibitory effect was much less as compared to the SAS cells.

### 2.2. Evaluation of the Effects of Dp44mT, DFO, and Defrasirox on NDRG1, NDRG2, NDRG3, Cyclin D1, and Maspin Expressions in SAS Cells

**Figure 2:**
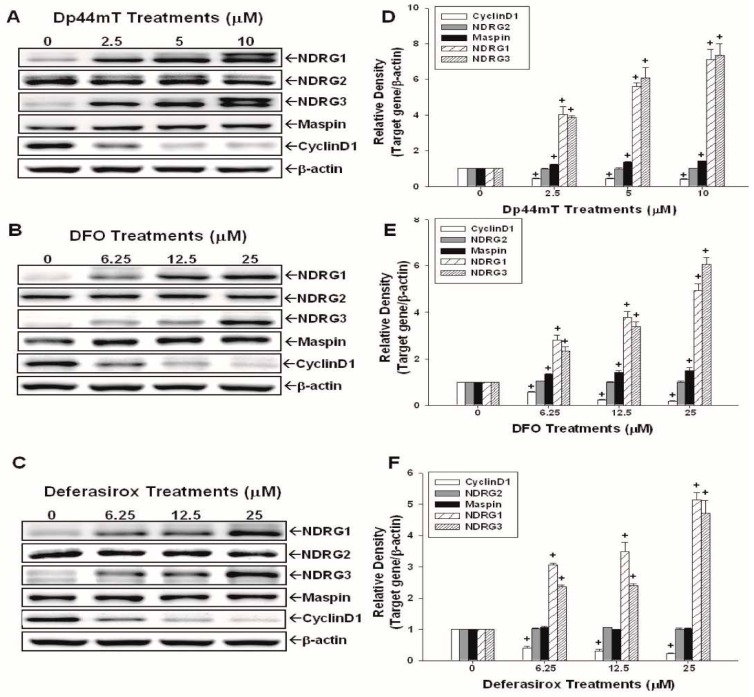
Effects of Dp44mT, DFO, and deferasirox on protein expressions of NDRG1, NDRG2, NDRG3, Maspin, and cyclin D1 in SAS cells. The SAS cells were treated with various concentrations of Dp44mt (**A**), DFO (**B**), and deferasirox (**C**) as indicated for 24 h, the expressions of NDRG1, NDRG2, NDRG3, Maspin, cyclin D1 proteins, and β-actin were determined by Western-blot assays. Data of quantitative analysis were expressed as the intensity of protein bands produced from the expressions of the target genes/β-actin (±SE; *n* = 3) relative to the control solvent-treated group (**D**–**F**), + *p* < 0.05.

Results of Western-blot assay and quantitative analysis illustrate that Dp44mT (Figure 2A,D), DFO (Figure 2B,E), and deferasirox (Figure 2C,F) treatments upregulated NDRG1 and NDRG3 protein levels in combination with the suppressions of cyclin D1 proteins in a dose-dependent manner. The quantitative analysis revealed that treatments of 10 µM Dp44mT induced a 7.1-fold increase of NDRG1 and a 7.3-fold increase of NDRG3 protein expressions (Figure 2D), and treatments of 25 µM DFO (Figure 2E) or deferasirox (Figure 2F) produced an increase by 4- to 4.5-fold in NDRG1 and a 5- to 6-fold in NDRG3 protein expressions, respectively, in SAS cells as compared to the control group. Dp44mT and DFO treatments also upregulated Maspin expression, while deferasirox treatments did not influence Maspin expression in SAS cells. Interestingly, none of Dp44mT, DFO, and deferasirox treatments significantly affected NDRG2 protein level in SAS cells.

### 2.3. Evaluation of the Effects of Dp44mT, DFO, and Defrasirox on NDRG1, NDRG2, NDRG3, Cyclin D1, and Maspin Expressions in OECM-1 Cells

**Figure 3:**
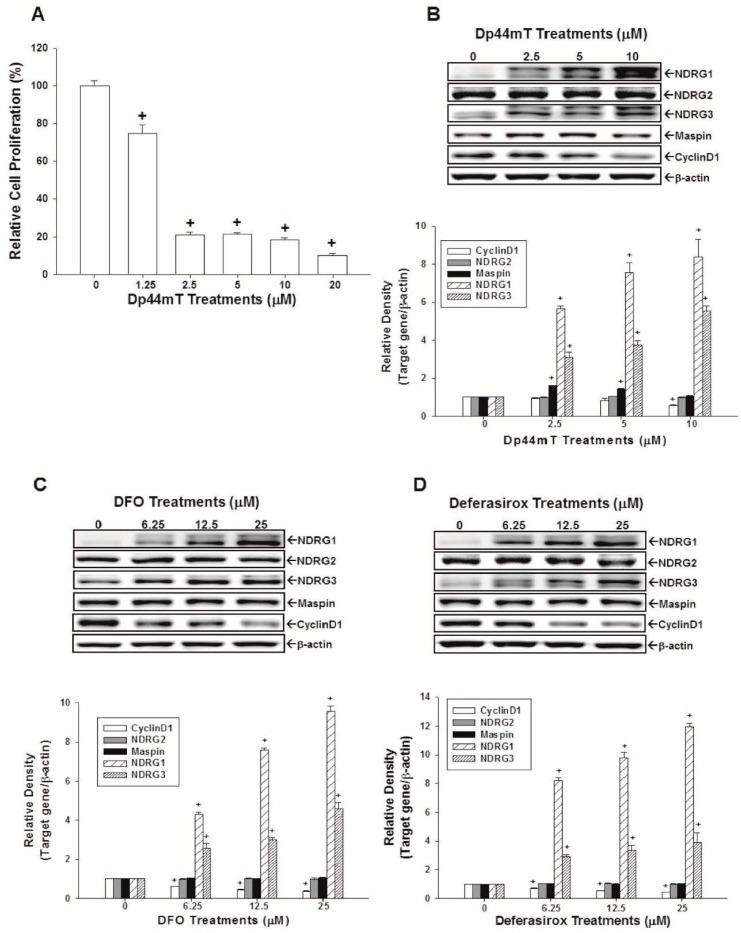
Anti-proliferative effects of Dp44mT and the effects of Dp44mT, DFO, and deferasirox on protein expressions of NDRG1, NDRG2, NDRG3, Maspin, and cyclin D1 in OECM-1 cells. (**A**) OECM-1 cells were treated with various concentrations of Dp44mt as indicated for 24 h and growth inhibitory effect was determined by the CyQUANT cell proliferation assay. The data shown in each bar chart represent the mean percentage ± SE of cells in each dose of the iron chelators treatment and are compared with the control solvent-treated group (*n* = 8). The OECM-1 cells were treated with various concentrations of Dp44mt (**B**), DFO (**C**), and deferasirox (**D**) as indicated for 24 h, the expressions of NDRG1, NDRG2, NDRG3, Maspin, cyclin D1 proteins, and β-actin were determined by Western-blot assays. Data of quantitative analysis were expressed as the intensity of protein bands produced from the expressions of the target genes/β-actin (±SE; *n* = 3) relative to the control solvent-treated group, + *p* < 0.05.

The CyQUANT cell proliferation assay reveals that Dp44mT significantly inhibited the growth of OECM-1 cells in a dose-dependent manner (Figure 3A). The protein levels of NDRG1 and NDRG3 were increased after treatments of Dp44mT (Figure 3B), DFO (Figure 3C), and deferasirox (Figure 3D), together with a decrease of cyclin D1 protein expression in OECM-1 cells. The quantitative analysis revealed that treatments of 10 µM Dp44mT significantly increased NDRG1 protein levels by 8.3-fold and NDRG3 protein levels by 5.3-fold (Figure 3B), and treatments of 25 µM DFO (Figure 3C) or deferasirox (Figure 3D) led to a 9.5- to 11.9-fold increase of NDRG1 and 4- to 4.6-fold increase of NDRG3 protein levels, respectively, in OECM-1 cells. Maspin expression in OECM-1 cells was increased by 2.5 and 5 µM Dp44mT treatment (Figure 3A).

### 2.4. NDRG3 Knockdown Enhanced OECM-1 Cells Proliferation and Tumor Growth in Vivo

**Figure 4:**
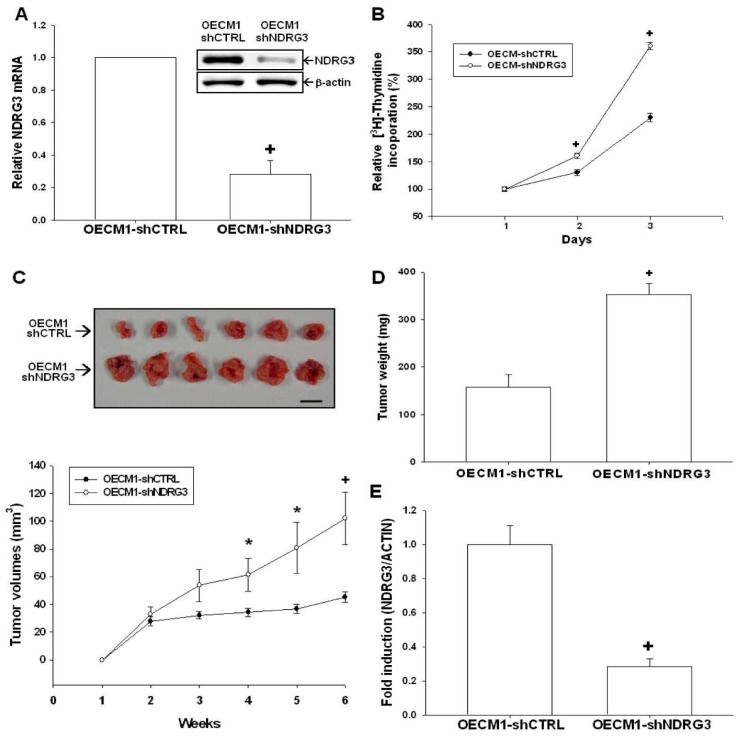
Knockdown of NDRG3 enhanced OECM-1 cell growth in vitro and in vivo xenograft mouse model. (**A**) The expressions of NDRG3 in mock-knockdown OECM-1 (OECM1-shCTRL) and NDRG3 knockdown OECM-1 (OECM1-shNDRG3) cells were determined by Western-blot (**top**) and RT-qPCR (**bottom**) assays. Data were expressed as mean (±SE; *n* = 3) of the NDRG3 mRNA levels in relation to the OECM1-shCTRL cell group; (**B**) Proliferations of OECM1-shCTRL (●) and OECM1-shNDRG3 (○) cells were determined by ^3^H-thymidine incorporation assays. Each point on the curve represented the mean-percentage (± SE; *n* = 6) of ^3^H-thymidine incorporated into the OECM-shNDRG3 cells relative to the OECM-shCTRL cells; (**C**) Nude mice were inoculated subcutaneously with OECM1-shCTRL (*n* = 6) or OECM1-shNDRG3 cells (*n* = 6) for 6 weeks. Values represented tumor size (mean ± SE) in mm^3^ of OECM1-shCTRL (●) and OECM1-shNDRG3 (○) groups (**bottom**) and photograph of the representative xenograph tumors after mice were sacrificed. Scale bar, 1 cm (**top**); (**D**) Comparison of tumor weight from animals with subcutaneously injected with OECM1-shCTRL or OECM1-shNDRG3 cells. The data were presented as mean (±SE) of tumor weight in milligrams; (**E**) The expressions of the NDRG3 gene in the xenograft tumor tissues of the OECM1-shCTRL and OECM1-shNDRG3 groups were determined using RT-qPCR assays. Data were presented as mean fold-induction of the mRNA levels of target gene (±SE) relative to the mock-transfected xenograft group, * *p* < 0.05, + *p* < 0.01.

To evaluate the biological role of NDRG3 in OSCC, we knocked down *NDRG3* in OECM-1 cells (OECM1-shNDRG3). The expressions of NDRG3 in selected clones were determined by Western-blot (Figure 4A, top) and RT-qPCR (real time quantitative polymerase chain reaction) (Figure 4A, bottom) assays. The results of ^3^H-thymidine incorporation assay reveals that knockdown of NDRG3 attenuated OECM-1 cell growth as OECM1-shNDRG3 cells had a much lower cell proliferative rate than OECM1-shCTRL cells (Figure 4B). The xenograft animal study demonstrates that OECM1-shNDRG3 cells-generated tumors grew more rapidly than those from OECM1-shCTRL cells. After 6 weeks of growth, the tumors derived from OECM1-shNDRG3 cells were found to have a 2.25-fold increase in average tumor size (102.01 ± 19.07 mm^3^ vs. 45.25 ± 3.68 mm^3^, Figure 4C) and a 2.23-fold increase in average tumor weight (353 ± 22.9 mg vs. 157.93 ± 25.8 mg, Figure 4D) as compared with the OECM1-shCTRL group. The expressions of *NDRG3* in the tumors were further measured by RT-qPCR assays (Figure 4E) which proved that *NDRG3* mRNA expression was much lower in the OECM1-shNDRG3 cell-generated tumors as compared to OECM1-shNDRG3 cell-generated tumors.

### 2.5. Dp44mT Inhibited SAS Cell Growth in Vivo

**Figure 5:**
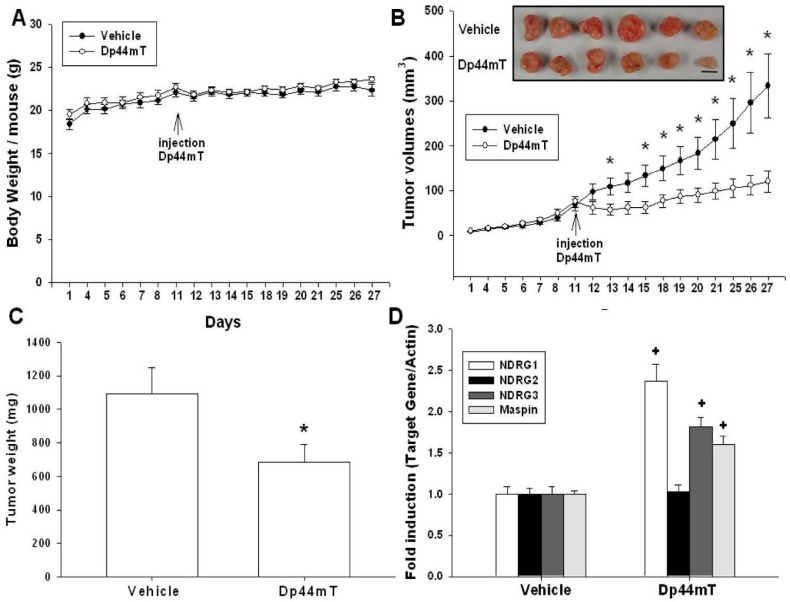
Dp44mT inhibited growth of SAS cells in xenograft mouse model. When tumor volumes reached 70 mm^3^ (day 11) after subcutaneous implantation of SAS cells, nude mice received vehicle (*n* = 6) or Dp44mT (0.5 mg/kg; *n* = 6) intravenously once per day, 5 days/week. (**A**) The average body weight (mean ± SE) after cell inoculation and during treatment; (**B**) Mice were sacrificed and tumors were collected at the 27th day after cell inoculation, and values represented tumor size (mean ± SE) in mm^3^ of vehicle treated (●) and Dp44mT treated (○) groups (**bottom**). Photograph of the representative xenograph tumors after mice were sacrificed. Scale bar, 1 cm (**top**); (**C**) Comparison of tumor weight from two animal groups. The data were presented as mean (±SE) of tumor weight in milligram; (**D**) The expressions of the *NDRG1*, *2*, *3*, and *Maspin* genes in the tumor tissues of the two groups of animals were determined by RT-qPCR assays. Data were presented as mean fold-induction of the mRNA levels of the target genes (±SE) relative to the vehicle treated xenograft group, * *p* < 0.05, + *p* < 0.01.

To evaluate the antitumor effects of Dp44mT on OSCC cells in vivo, a xenograft animal model was applied. After solid tumors were established (about 70 mm^3^ at day 11 following SAS cell inoculation), Dp44mT (0.5 mg/kg) was administered intravenously once daily for 5 days a week, and 17 days totally. During the treatment period, the average body weight of the Dp44mT-treated mice was not significantly different from vehicle-treated mice (19.53 ± 0.57 to 23.6 ± 0.34 g in Dp44mT-treated group versus 18.42 ± 0.39 to 22.34 ± 0.67 g in vehicle-treated group, Figure 5A). After 17 days of treatment, the tumors derived from Dp44mT-treated mice present reductions of 63.81% in tumor size (120.61 ± 24.08 mm^3^ vs. 333.31 ± 71.11 mm^3^, Figure 5B) and 37.32% in tumor weight (683.80 ± 109.06 mg vs. 1090.98 ± 155.04 mg, Figure 5C), as compared with tumors from vehicle-treated animals. Furthermore, results of RT-qPCR assays (Figure 5D) show that mRNA of NDRG1, NDRG3, and Maspin was increased in the xenografted tumors from Dp44mT-treated mice.

## 3. Discussion

Treatment of oral cancer is usually multidisciplinary, which includes surgical resection, radiation, and chemotherapy [4,28]. Even though, for patients with OSCC, the overall 5-year survival rate is only around 50%. Moreover, the uncomfortable side effects generated from radiation and chemotherapy, and functional and cosmetic problems after surgery further complicate OSCC treatment. Thus, it is urgently necessary to explore new therapeutic molecular targets and agents for OSCC treatment.

A number of cancers can be inhibited by iron chelators in terms of cell growth, and several antitumor mechanisms of iron chelators have been well documented [8]. However, the antitumor activities of iron chelators in OSCC have not yet been evaluated. In this study, we showed the antitumor effects of iron chelators on OSCC in vitro and in vivo, and found some iron chelator downstream genes in OSCC cells.

NDRG1 has been shown to have numeral effects on cancer cells, such as pro-differentiation, cell cycle arrest, and metastasis attenuation, and, thus, been inferred as a tumor suppressor gene in OSCC cells [16]. Cyclin D1, a key regulator of cell proliferation, has been reported that its gene amplification and/or protein overexpression increased the malignant transformation risk in oral cells [29]. Studies further indicated that cyclin D1 overexpression occurred early in the oral tumorigenesis process and significantly associated with advanced tumor stages [30,31]. Our results reveal that Dp44mT, DFO, and deferasirox treatments significantly inhibited cell growth and caused cell cycle G1 phase arrest (Figure 1). The Western-blot assays also show that these three iron chelators increased NDRG1 expression while decreasing cyclin D1 expression in OSCC cells (Figure 2 and Figure 3), which were consistent with previous in vitro and in vivo studies [7,18,32,33,34,35,36].

We further evaluated the effects of iron chelators on NDRG2, NDRG3, and Maspin expressions in OSCC cells. Although one recent study showed that Dp44mT inhibited the invasion and metastasis in hepatocellular carcinoma cells by increasing NDRG2 expression [19], our result shows that treatment of iron chelators did not affect NDRG2 expression in SAS and OECM-1 cells. We further demonstrated that incubation of SAS and OECM-1 cells with iron chelators enhanced the expressions of NDRG3 dose-dependently (Figure 2 and Figure 3). Maspin is a non-inhibitory serpin and has been reported as a potential tumor suppressor gene in several cancers including OSCC [24,25,26,27,37,38]. Our results indicate that Dp44mT treatment enhanced Maspin expressions in SAS and OECM-1 cells; however, DFO increased Maspin expressions only in SAS cells, and deferasirox treatment did not affect Maspin expressions in both OSCC cell lines. It has been reported that different classes of ion chelators may affect different target genes in various cancer cells [35], which is consistent with our current finding. To our knowledge, this is the first report indicating that Dp44mT treatment increased the expressions of NDRG3 and Maspin genes in OSCC cells. Regarding iron chelators′ effect on *N*-myc gene expression, the upstream gene of NDRGs, iron chelators have been shown to inhibit *N*-myc expression in neuroblastoma cells [39]. Regarding OSCC cells, no any reports have been published yet. Further studies are needed to elucidate whether iron chelators could affect *N*-myc expression in OSCC cells or not.

Although previous studies indicated that NDRG3 overexpression might increase cell growth, angiogenesis, tumorigenesis, and metastasis in prostate and liver cancers [21,22,23], the function of NDRG3 is still not well understood in OSCC. Our results demonstrate that NDRG3-knocked down OECM-1 cells presented a significantly higher growth rate compared to that of mock-transfected OECM-1 cells. In addition, the xenograft animal study further shows that knockdown of NDRG3 enhanced the tumor growth of OECM-1 cells in vivo (Figure 4). Our study is the first report suggesting that NDRG3 may function as a tumor suppressor gene in OSCC cells.

To confirm in vivo antitumor effects of iron chelators on OSCC cells, SAS cells were then xenografted in nude mice. Our xenograft animal study (Figure 5) demonstrates that tumor growth rate of Dp44mT-treated group was lower than that of vehicle-treated group. The applied dose of Dp44mT (0.5 mg/kg/day) showed no significant influence on mice body weight, indicating the safety of Dp44mT application in vivo under this dose. Our results were supported by previous studies which demonstrated the Dp44mT was an effective regimen for cancer treatment in vivo, including pancreatic, lung, and hepatic cancers and did not affect the body weight of animals [6,7,9,18,19,35]. About deferasirox, it has been shown to inhibit esophageal cancer and lung cancer growth in vivo [7,40]. DFO has been deemed as a promising agent for brain tumor therapy [41]. The results of an RT-qPCR assay further show that mRNA levels of NDRG1, NDRG3, and Maspin of Dp44mT-treated xenografted tumors were upregulated as compared with the vehicle-treated group (Figure 5D), indicating that NDRG1, NDRG3, and Maspin expressions were indeed repressed by Dp44mT in vivo, which is also consistent with the findings shown in Figure 2 and Figure 3.

## 4. Materials and Methods

### 4.1. Cell Culture and Chemicals

The human oral squamous carcinoma cancer cell lines and OECM-1 cells were derived from OSCC patients of Taiwan, and the SAS cells were obtained from Japanese Collection of Research Bioresources (JCRB, Tokyo, Japan). Both cell lines were maintained in RPMI-1640 medium (Life Technologies, Rockville, MD, USA) with 10% fetal calf serum (FCS; HyClone, Logan, UT, USA) as previously described [16,42].The identity of the cells was confirmed by short tandem repeat (STR-PCR) analysis (Genelabs, Taipei, Taiwan). Dp44mT and DFO were purchased from Sigma-Aldrich Co. (St. Louis, MO, USA). Deferasirox was purchased from Santa Cruz Biotechnology (Santa Cruz, CA, USA). All chemicals were dissolved in the suggested solvent, according to the manufacturer′s instructions. The normal human epithelial cells, HaCaT cells, was a gift from Jong-Hwei S. Pang.

### 4.2. Knockdown NDRG3

OECM-1 cells were transduced with *NDRG3* small hairpin RNA lentiviral particles (sc-40759-V; Santa Cruz Biotechnology) as the NDRG3-knockdown (OECM1-shNDRG3) cells, and with control small hairpin RNA lentiviral particles (sc-10808-V, Santa Cruz Biotechnology) as the mock-transfected OECM-1 (OECM1-shCTRL) cells. Two days after transduction, the OECM1-shNDRG3 and OECM1-shCTRL cells were selected with puromycin dihydrochloride (4 µg/mL) for at least 4 generations.

### 4.3. Colony Formation Assay

Colony formation assay was used to assess the anti-growth efficacy of Dp44mT on SAS cells. The SAS cells were cultured in a 6-well plate (200 cells/well) and then exposed to different concentrations (0–20 µM) of Dp44mT for 24 h. After removal of the supernatant, cells continued to incubate in RPMI medium with 10% FCS for another 7 days. The colonies were fixated and counted manually after staining with 0.5% crystal violet as previously described [43].

### 4.4. Cell Proliferation Assay

Proliferation of cells after treated with iron chelators were measured using a CyQUANT cell proliferation assay kit as previous described [44]. Cell proliferation of OECM1-shCTRL or OECM1-shNDRG3 cells was determined with a ^3^H-thymidine incorporation assay as previously described [45]. In brief, cells were cultured in a 6-well plate (1 × 10^4^/well) for 24 h, and then added ^3^H-thymidine to each well (0.5 µCi/mL) and cultured at 37 °C for 4 h. After being washed and lysed, the solubilized cell solution was mixed with scintillation cocktail before being counted in a liquid scintillation analyzer (Packard BioScience, Downers Grove, IL, USA).

### 4.5. Flow Cytometry

In response to the Dp44mT treatment, SAS cells (1 × 10^5^/flask) were serum starved for 24 h and then cultured in medium with 10% FCS and various concentrations (0–20 µM) of Dp44mT for another 24 h. The cells were trypsinized, fixed in ethanol, digested in Triton X-100 and ribonuclease, and stained with propidium iodide. We sorted 1 × 10^4^ cells in each dosage to analyze the cell cycle distribution with the FACS-Calibur Cytometer and CellQuestPro Software (BD Biosciences, San Jose, CA, USA); the data were analyzed using ModFit LT Mac 3.0 Software as previously described [42].

### 4.6. Western-Blot Assay

Concentrations of protein in aliquot samples were measured using the Pierce BCA protein assay kit (Thermo Fisher Scientific Inc., Waltham, MA, USA). Equal quantities of cell extracts were separated on a 10% SDS-PAGE gel, transferred and analyzed by the Western lightning plus-ECL detection system (Perkin Elmer, Inc., Waltham, MA, USA). Membranes were probed overnight at 4 °C with antibodies for NDRG1 (42-6200; Invitrogen, Carlsbad, CA, USA), NDRG2 (ab169775; Abcam, Cambridge, UK), NDRG3 (ab131266; Abcam), cyclin D1 (DCS6, Cell Signaling, Danvers, MA, USA), and Maspin (554292; BD Biosciences). The β-actin (MAB1501; Millipore, Temecula, CA, USA) was used as the internal positive control. Images were viewed by using ChemiGenius image capture system (Syngene, Cambridge, UK) and the intensities of the different bands were analyzed by using GeneTools program of ChemiGenius (Syngene).

### 4.7. Real-Time Reverse Transcription-Polymerase Chain Reaction (RT-qPCR)

Total RNA from cells was isolated by using Trizol reagent, cDNA was synthesized, and real-time polymerase chain reaction (qPCR) was performed as described before [37]. FAM dye-labeled TaqMan MGB probes for human *NDRG1* (Hs00608387_m1), *NDRG2* (Hs01045115_m1), *NDRG3* (Hs00223890_m1), *Maspin* (Hs00985283_m1), and β*-actin* (Hs01060665_g1) were purchased from Applied Biosystems (Foster City, CA, USA).

### 4.8. Xenograft Tumors of OSCC Cells in Nude Mice

All animal experiments met the Guide for Laboratory Animal Facilities and Care as promulgated by Council of Agriculture Executive Yuan, Taiwan. The protocol was approved by the Mackay Memorial Hospital Animal Research Committee (Permit Number: MMH-A-S-103-32). All surgical procedures were carried out under anesthesia, and all efforts were made to minimize suffering. Twenty-four 4-week-old male BALB/cAnN-Foxn1^NU^ mice were used in this study. Mice were kept in a barrier facility under HEPA filtration and animal health was monitored twice-weekly during experiment. Each mouse was anesthetized intraperitoneally with a 100 µL injection of a mixture of 2.5% tribromoethanol and 2.5% tert-amyl alcohol in Tris buffer solution. To assess the effect of NDRG3 in OECM-1 cells in vivo, equal volumes of OECM1-shCTRL or OECM1-shNDRG3 cells (5.7 × 10^6^/100 µL) were injected subcutaneously. The growth of xenograft was measured by using vernier calipers at 3-day intervals. To evaluate the antitumor effect of iron chelators in OSCC cells, the male nude mice (BALB/cAnN-Foxn1, 4 weeks old) were anesthetized intraperitoneally and equal volumes of SAS cells (4.6 × 10^6^/100 µL) were injected subcutaneously. On day 11, the mice with xenograft tumor volumes (≥70 mm^3^) were randomized into two groups. Dp44mT (0.5 mg/kg) was dissolved in 15% propylene glycol in 0.9% saline as described in detail previously [6,9]. The Dp44mT group was administered intravenously via the tail vein once daily for 5 days a week (total 17 days). Controlled mice were treated with vehicle only (vehicle treated group). Tumor volume and mouse body weight were measured five times weekly. Tumor volume was calculated as π/6 × larger diameter × (smaller diameter)^2^ as previously described [42].

### 4.9. Statistical Analysis

Results are expressed as the mean ± SE of at least three independent replications of each experiment. Statistical significance was determined using Student′s paired *t-*test and one way ANOVA using the SigmaStat program for Windows, version 2.03 (SPSS Inc., Chicago, IL, USA). Significance was established at a *p* value less than 0.05 (* *p* < 0.05) or 0.01 (+ *p* < 0.01).

## 5. Conclusions

Iron chelators exhibited a potent anti-growth effect on OSCC cells, with Dp44mT being further shown to inhibit SAS cell growth in vivo through a xenograft animal model, suggesting that iron chelators have potential as new regimens for OSCC cancer treatment. NDRG3 overexpression exhibited an inhibitory effect on cell proliferation of OSCC cells in vitro and in vivo. The growth inhibition ofDP44mT on OSCC cells could partly be attributed to the induction of NDRG1, NDRG3, and Maspin and repression of cyclin D1.

## Supplementary Materials

Supplementary materials can be found at www.mdpi.com/1422-0067/17/9/1435/s1.
