# Supplementary material for: The Iron Chelator, Dp44mT, Effectively Inhibits Human Oral Squamous Cell Carcinoma Cell Growth in Vitro and in Vivo
Source: Int J Mol Sci. 2016 Aug 31;17(9):1435. doi: 10.3390/ijms17091435 (PMC5037714; doi:10.3390/ijms17091435)
Supplement: Supplementary file 1 [file ijms-17-01435-s001.pdf]

Supplementary Material: The Iron Chelator, Dp44mT, Effectively Inhibits Human Oral Squamous Cell Carcinoma Cell Growth in Vitro and in Vivo

Jehn-Chuan Lee, Kun-Chun Chiang, Tsui-Hsia Feng, Yu-Jen Chen, Sung-Ting Chuang, Ke-Hung Tsui, Li-Chuan Chung and Horng-Heng Juang

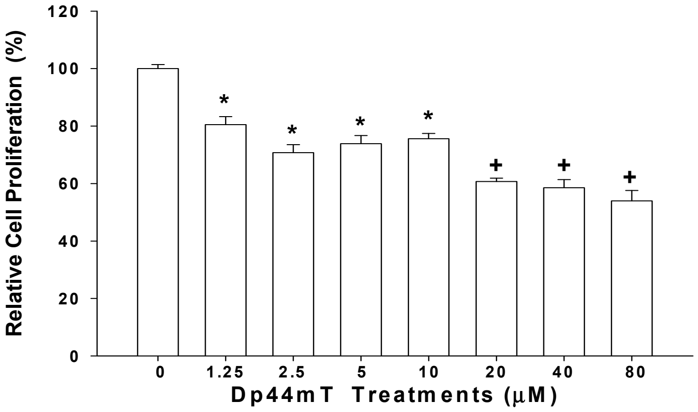

Figure S1. Anti-proliferative effects of Dp44mT o HaCaT cells. The HaCaT cells were treated with various concentrations of Dp44mt for 24 h and cell growth was measured by the CyQUANT cell proliferation assay. The data shown in each bar chart represented the mean percentage ± SE of cells in each dose of the iron chelator treatment and were compared with the control solvent-treated group ($n = 8$) (* $p < 0.05$, + $p < 0.01$).

As treated HaCaT cells with varied doses of Dp44mT for 24 h, although the cell proliferation was still repressed, however, the extent of inhibition was much less than that of cancer cells.
